# The real-world selection of first-line systemic therapy regimen for metastatic gastroenteropancreatic neuroendocrine neoplasm in Japan

**DOI:** 10.1038/s41598-022-22718-8

**Published:** 2022-10-20

**Authors:** Shun Yamamoto, Naoki Sakakibara, Hidekazu Hirano, Chigusa Morizane, Yoshitaka Honma, Susumu Hijioka, Takuji Okusaka, Takahiro Higashi, Akira Kawai

**Affiliations:** 1 Department of Head and Neck, Esophageal Medical Oncology, National Cancer Center Hospital, Tokyo, Japan; 2 Department of Gastrointestinal Medical Oncology, National Cancer Center Hospital, Tokyo, Japan; 3 Division of Health Services Research, Institute for Cancer Control, National Cancer Center, Tokyo, Japan; 4 Department of Hepatobiliary and Pancreatic Oncology, National Cancer Center Hospital, 5-1-1 Tsukiji, Chuo-Ku, Tokyo, 1040045 Japan; 5 Department of Musculoskeletal Oncology and Rehabilitation, National Cancer Center Hospital, Tokyo, Japan

**Keywords:** Cancer, Medical research, Oncology

## Abstract

In November 2013, the first edition of evidence-based guidelines for treatment of gastroenteropancreatic neuroendocrine neoplasm (GEP-NEN) was published in Japan. However, whether medical practitioners have adopted the first-line regimens recommended for metastatic GEP-NEN in clinical practice is not yet known. The purpose of this study was to identify which first-line systemic therapy regimens have been selected and the proportion of cases that are adherent to the guidelines (i.e., number of patients receiving recommended therapy/total number of patients). We combined hospital-based cancer registry data and insurance claims-equivalent data for patients with GEP-NEN treated between January 2013 and December 2014 and extracted those with metastatic GEP-NEN who received systemic therapy. The proportions that were adherent with the guideline were calculated according to tumor classification (neuroendocrine tumor [NET] or neuroendocrine carcinoma [NEC]), primary site (gastrointestinal or pancreatic), and hospital volume (high, medium, or low). The study included 109 patients with GEP-NET and 424 with GEP-NEC. Overall, guideline-adherent treatment was provided in only 54.8% of cases (58.1% for gastrointestinal NET, 63.6% for pancreatic NET, 56.6% for gastrointestinal NEC, and 44.9% for pancreatic NEC). The recommended therapy for GEP-NET was used in 16.5% of patients with GEP-NEC, and 21.5% received fluoropyrimidine-containing chemotherapy. This report is the first to describe real-world selection of first-line regimens for metastatic GEP-NEN. About half of all these patients received systemic therapy that was not recommended in the guidelines.

## Introduction

Neuroendocrine neoplasm (NEN) is a rare heterogeneous tumor arising from neuroendocrine cells in the diffuse endocrine system. Although NENs can occur throughout the body, a gastroenteropancreatic (GEP) origin is most common. According to the World Health Organization classification^1,2^, NEN is broadly divided into two types: low-grade neuroendocrine tumor (NET) and high-grade neuroendocrine carcinoma (NEC). In the US, the annual incidence of GEP-NEN has been reported to be 3.56 per 100,000 population, with increasing trends in both incidence and prevalence^3^; in Japan, the annual adjusted incidence of GEP-NEN is reportedly 3.53 per 100,000^4,5^.

Despite the rarity of GEP-NEN, clinical guidelines have been published by the European Neuroendocrine Tumor Society (ENETS)^6,7^ and the National Comprehensive Cancer Network (NCCN)^8^ to promote standardization of clinical practice based on evidence obtained from clinical research. In November 2013, the Japan Neuroendocrine Tumor Society (JNETS) also published clinical guidelines for GEP-NEN for the same purpose. These guidelines recommend use of a somatostatin analog (octreotide or lanreotide), a molecular targeted agent (everolimus or sunitinib), and streptozocin for metastatic GEP-NET and a platinum-containing regimen (platinum plus etoposide or irinotecan) for metastatic GEP-NEC.

However, whether the first-line regimens recommended in the guidelines for patients with metastatic GEP-NEN have been adopted in clinical practice in Japan remains unclear. Therefore, the purpose of this study was to clarify the real-world selection of first-line systemic therapy for metastatic GEP-NEN using both hospital-based cancer registry^9^ and patient insurance claims-equivalent data.

## Materials and methods

### Data source

This study is a secondary analysis of health insurance claims data linked with the hospital-based cancer registry of the project examining quality assessments and improvements^10^. This project used patient insurance claims-equivalent data that had been linked to data from hospital-based registries since 2011. All cancer care hospitals designated by the Ministry of Health, Labour and Welfare were invited to participate in the project, as were non-designated cancer care hospitals that played similar roles and had submitted their hospital-based registry data to the National Cancer Center for its annual statistical report. Designated cancer care hospitals play a central role in cancer care across communities in Japan^11^. As a condition for receiving this designation, these hospitals are required to provide standard treatment, including surgery, chemotherapy, and radiation, for cancer patients.

The data from the hospital-based cancer registries included the following information on GEP-NEN patients: (1) clinical profiles, such as birth date, sex, tumor sites, and histology codes according to the International Classification of Diseases for Oncology, third edition; (2) clinical tumor-node-metastasis (TNM) staging based on the Union for International Cancer Control (UICC); (3) diagnosis month; and (4) details of first-line systemic therapy. The insurance claims-equivalent data contained the details of care for each patient, such as all the procedures and prescriptions provided to the patient with the corresponding dates in a manner like that used for fee-for-service insurance claims. We have referred to this data as “claims-equivalent” because the data points themselves are not used for reimbursement but are instead collected to monitor the effects of changes to reimbursement rules on care utilization. These two data sources were linked and anonymized according to hospital before data submission to the National Cancer Center.

In this study, data from the linked databases that were submitted to the National Cancer Center in 2013 and 2014 were used. Overall, 309 hospitals joined the project, with 240 joining in 2012 (234 designated and 6 non-designated cancer care hospitals) and 297 joining in 2013 (290 designated and 7 non-designated cancer care hospitals). Information on 858,565 cancers (371,609 in 2012 and 486,956 in 2013) was collected. In this study using a secondary data, informed consent was obtained in the form of opt-out on the website.

### Study population

From the linked data, we extracted information on patients diagnosed as having metastatic GEP-NET (ICD-O-3 histology codes 8151/3, 8152/3, 8153/3, 8155/3, 8156/3, 8240/3, 8241/3, 8242/3, 8249/3, and 8150/3) and GEP-NEC (ICD-O-3 histology codes 8002/3, 8012/3, 8013/3, 8014/3, 8041/3, 8042/3, 8043/3, 8044/3, 8045/3, 8244/3, and 8246/3).

### Definition of first-line systemic therapy regimens recommended in the guidelines

First, we identified the systemic therapy regimen that was administered on schedule and closest to the month of diagnosis with GEP-NET or GEP-NEC; this regimen was defined as the first-line systemic therapy regimen. If another anti-cancer drug was administered within 14 days of the first dose, the treatment was considered to be a combination systemic therapy, and the combined therapy was defined as the first-line systemic therapy regimen.

Second, after considering the ENETS guidelines^6,7^, the NCCN guidelines^8^, and the JNET guidelines^12^, we defined octreotide (approved in 2011)^13^, lanreotide (approved in 2017)^14^, everolimus (approved in 2016)^15,16^ and streptozocin (approved in 2014)^17^ for metastatic gastrointestinal (GI)-NETs; octoreotide (approved in 2011)^13^, lanreotide (approved in 2017)^14^, everolimus (approved in 2011) ^15,16^, sunitinib (approved in 2012)^18^, and streptozocin (approved in 2014)^17^ for metastatic pancreatic (P)-NETs; and etoposide plus cisplatin/carboplatin and irinotecan plus cisplatin/carboplatin^19,20^ for metastatic GEP-NECs as the first-line standard systemic therapy regimens recommended in the guidelines (Table 1).

**Table 1:** Definitions of guideline-adherent first-line systemic therapy regimens used in patients with GEP-NEN in this study.

Tumor classification	Recommended regimen
GI-NET	Octreotide, everolimus, streptozocin
P-NET	Octreotide, everolimus, sunitinib, streptozocin
GI-NEC	Etoposide plus cisplatin/carboplatin, irinotecan plus cisplatin/carboplatin
P-NEC	Etoposide plus cisplatin/carboplatin, irinotecan plus cisplatin/carboplatin

*GI-NEC* gastrointestinal neuroendocrine carcinoma, *GI-NET* gastrointestinal neuroendocrine tumor, *P-NEC* pancreatic neuroendocrine carcinoma, *P-NET* pancreatic neuroendocrine tumor.

### Statistical analysis

To investigate the relationships between the selection of a first-line systemic therapy regimen and the hospital volume, we defined hospital volume based on the annual number of patients visiting each hospital. If a hospital participated in this project for two years, the mean annual number of patients from 2013 to 2014 was used as the annual patient number. Based on the annual patient number, hospital volume was then divided into three groups (high-volume [the highest tertile group], medium-volum [the middle tertile group] and low-volume [the lowest tertile group]). The proportion of cases with guideline adherence was defined as the number of patients who received systemic therapies recommended in the guidelines (Table 1) divided by the number of all eligible patients who received systemic therapies. For example, the guideline adherence proportion of GI-NET patients in high-volume hospitals was defined as the number of GI-NET patients who received octreotide, everolimus, or streptozocin in high-volume hospitals divided by the number of all GI-NET patients who received systemic therapies in high-volume hospitals.

A descriptive analysis was performed for the baseline patient characteristics according to hospital volume. The groups were then compared using a Fisher exact test for categorical variables. We calculated the proportion of guideline adherence according to hospital volume, tumor classification, primary sites and year (2013 and 2014; corresponding to before and after publication of the Japanese guidelines). All the tests evaluating significance were two-tailed, with the alpha value was at 0.05. All statistical analyses were performed using STATA version 14.2 software.

### Ethical statement

This study was conducted in accordance with the Declaration of Helsinki, and this study was approved by the institutional review boards of the National Cancer Center, Japan (2013-081).

## Results

**Figure 1:**
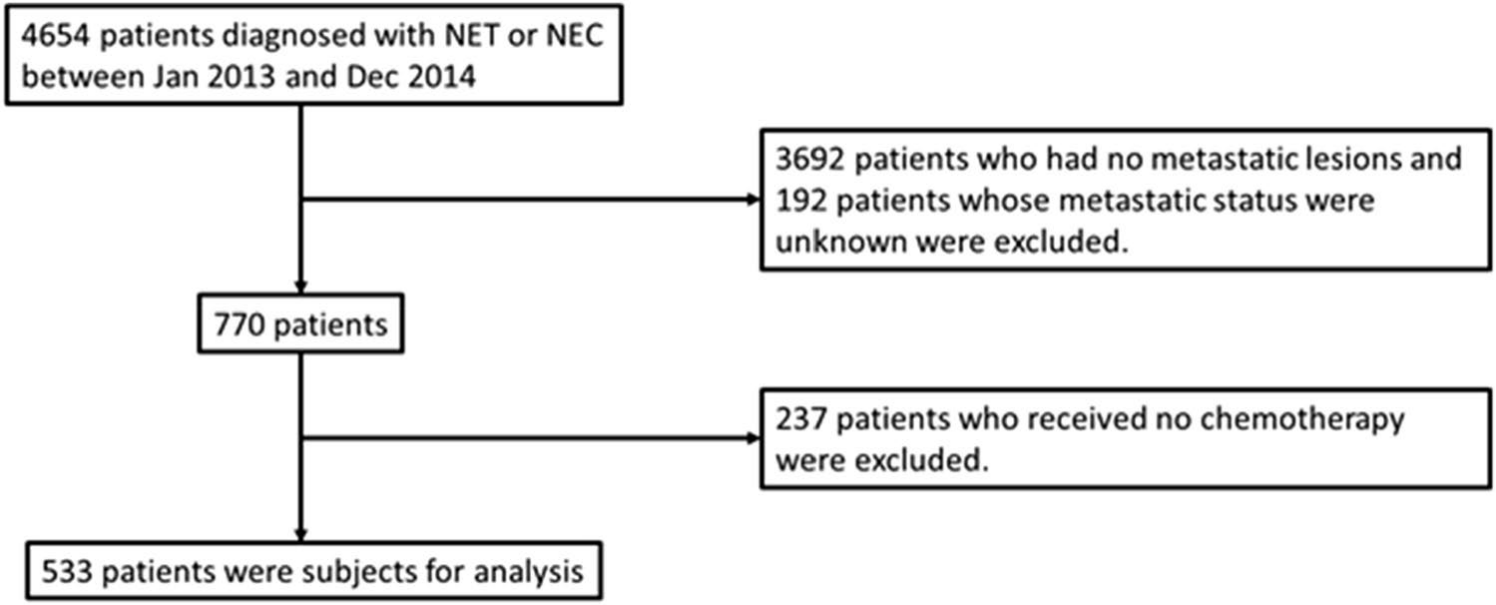
Flowchart for extraction of patients.

Figure 1 shows the flowchart for the selection of the dataset to be used for the analysis from the linked data. Of the 4654 patients who were diagnosed as having NEN in 2013–2014, 533 patients who were diagnosed as having metastatic GEP-NEN and had received first-line systemic therapy at 218 participating hospitals were extracted for the analysis. The patients’ characteristics are shown in Table 2, with subgroups classified according to hospital volume (high-volume: 2.5 ≤ patient/year, medium-volume: 1 < , < 2.5 patient/year, and low-volume: ≤ 1 patient/year). The median age of the metastatic GEP-NEN patients was 66 years, and 350 and 183 patients were male and female, respectively. The numbers of metastatic GEP-NET and GEP-NEC patients were 109 (20.5%) and 424 (79.5%), respectively. Forty-three patients (8.1%) and 66 patients (12.4%) had metastatic NETs with a gastrointestinal primary site and a pancreatic primary site, respectively. Two hundred and ninety-seven patients (55.7%) and 127 patients (23.7%) had metastatic NECs of the gastrointestinal tract and pancreas, respectively. Additionally, of 237 excluded patients who received no chemotherapy, 96 patients (40.5%) received surgical resections.

**Table 2:** Patient characteristics according to hospital volume.

Characteristics	All patients (%)	Hospital volume*		
		High (%)	Medium (%)	Low (%)
Patients, n	533 (100)	211 (100)	174 (100)	148 (100)
**Primary site**				
Gastrointestinal tract	340 (63.8)	118 (55.9)	119 (68.4)	103 (69.6)
Pancreas	193 (36.2)	93 (44.1)	55 (31.6)	45 (30.4)
**Tumor classification**				
NET	109 (20.5)	47 (22.3)	34 (19.5)	28 (18.9)
GI-NET	43 (8.1)	18 (8.5)	14 (8.0)	11 (7.4)
P-NET	66 (12.4)	29 (13.7)	20 (11.5)	17 (11.5)
NEC	424 (79.5)	164 (77.7)	140 (80.5)	120 (81.1)
GI-NEC	297 (55.7)	100 (47.4)	105 (60.3)	92 (62.2)
P-NEC	127 (23.8)	64 (30.3)	35 (20.1)	28 (18.9)
Median age (years)	66 (17–91)	65 (17–85)	66 (29–91)	66.5 (23–88)
**Sex**				
Male	350 (65.7)	133 (63.0)	120 (69.0)	97 (65.5)
Female	183 (34.4)	78 (37.0)	54 (31.0)	51 (34.5)
**Previous treatment**				
Surgery	101 (18.9)	26 (12.3)	48 (27.6)	27 (18.2)
Radiotherapy	18 (3.4)	8 (3.8)	9 (5.2)	1 (0.7)

*GI-NEC *gastrointestinal neuroendocrine carcinoma, *GI-NET *gastrointestinal neuroendocrine tumor, *P-NEC* pancreatic neuroendocrine carcinoma, *P-NET* pancreatic neuroendocrine tumor.

*Hospital volume was divided into three groups according to number of patients with GEP-NEN treated per year. The upper third of hospitals were designated as high-volume, the middle third as medium-volume, and the lower third as low-volume.

Table 3 shows the real-world selection of first-line systemic therapy regimens for the GEP-NET patients. Among 43 metastatic GI-NET patients, 58.1% received octreotide or everolimus as a guideline-adherent first-line systemic therapy. On the other hand, 11.6% of the patients received a platinum-containing regimen, and 14.0% received a fluoropyrimidine-containing regimen. Hospital volume tended to be associated with the guideline adherence proportion: 77.8% for high-volume hospitals, 50.0% for medium-volume hospitals, and 36.4% for low-volume hospitals (*p* = 0.065). When calculated according to year, the guideline adherence proportions were 58.8% (10/17) in 2013 and 57.7% (15/26) in 2014 (*p* = 1.000, Table 5).

**Table 3:** Real-world selection of first-line systemic therapy regimens for patients with GEP-NET according to hospital volume subgroup.

Regimens for GI-NET	All patients N = 43 (%)	High-volume n = 18 (%)	Medium-volume n = 14 (%)	Low-volume n = 11 (%)	*p *value
Octreotide	24 (55.8)	14 (77.8)	6 (42.9)	4 (36.4)	0.065
Everolimus	1 (2.3)	0 (0)	1 (7.1)	0 (0)	
Streptozocin	0 (0)	0 (0)	0 (0)	0 (0)	
Platinum-based	5 (11.6)	2 (11.1)	0 (0)	3 (27.3)	
Other*	13 (30.2)	2 (11.1)	7 (50.0)	4 (36.4)	
Guideline-adherent proportion	**25 (58.1)**	**14 (77.8)**	**7 (50.0)**	**4 (36.4)**	

Regimens for P-NET	All patients N = 66 (%)	High-volume n = 29 (%)	Medium-volume n = 20 (%)	Low-volume n = 17 (%)	
Octreotide	14 (21.2)	5 (17.2)	5 (25.0)	4 (23.5)	0.849
Everolimus	22 (33.3)	9 (31.0)	7 (35.0)	6 (35.3)	
Sunitinib	6 (9.1)	4 (13.8)	0 (0)	2 (11.8)	
Streptozocin	0 (0)	0 (0)	0 (0)	0 (0)	
Gemcitabine	4 (6.1)	1 (3.4)	0 (0)	3 (17.6)	
Other^†^	20 (30.3)	10 (34.5)	8 (40.0)	2 (11.8)	
Guideline-adherent proportion	**42 (63.6)**	**18 (62.1)**	**12 (60.0)**	**12 (70.6)**	

*GEP* gastroenteropancreatic; *GI-NET* gastrointestinal neuroendocrine tumor, *P-NET* pancreatic neuroendocrine tumor.

*Epirubicin, epirubicin + cyclophosphamide + fluorouracil, epirubicin + mitomycin, capecitabine, tegafur/uracil, octreotide + dacarbazine, octreotide + epirubicin + fluorouracil + mitomycin.

^†^Epirubicin, cisplatin, cisplatin + etoposide, cisplatin + irinotecan, S-1, epirubicin + octreotide, cisplatin + irinotecan + octreotide, gemcitabine + cisplatin + irinotecan + octreotide, gemcitabine + everolimus + octreotide, gemcitabine + fluorouracil + irinotecan + oxaliplatin, sunitinib + cisplatin.

Significant values are in bold.

Among 66 metastatic P-NET patients, 21.2% of the patients received octreotide, 33.3% received everolimus, and 9.1% received sunitinib as a guideline-adherent first-line systemic therapy. On the other hand, 6.1% of the patients received gemcitabine, and 16.7% received a platinum-containing regimen. The guideline adherence proportion was 63.6%. When evaluated according to hospital volume, these proportions were 62.1% for high-volume hospitals, 60.0% for medium-volume hospitals, and 70.6% for low-volume hospitals (*p* = 0.849). When calculated according to year, the guideline adherence proportions were 60.0% (15/25) in 2013 and 65.9% (27/41) in 2014 (*p* = 0.793, Table 5).

Table 4 shows the real-world selection of first-line systemic therapy regimens for GEP-NEC patients. Among 297 metastatic GI-NEC patients, 22.6% received etoposide and cisplatin/carboplatin, and 34.0% received irinotecan and cisplatin/carboplatin as a guideline-adherent first-line systemic therapy. Specifically, 7.4% of the patients received octreotide or everolimus and 14.5% of the patients received fluoropyrimidine-based chemotherapy. The guideline adherence proportion was 56.6%. A significant difference in guideline adherence proportions according to hospital volume was observed: 75.0% for high-volume hospitals, 51.4% for medium-volume hospitals, and 42.4% for low-volume hospitals (*p* < 0.001). When calculated according to year, the guideline adherence proportions were 48.5% (65/134) in 2013 and 61.3% (103/163) in 2014 (*p* = 0.013, Table 5).

**Table 4:** Real-world selection of first-line chemotherapy regimens for patients with GEP-NEC according to hospital volume.

Regimens for GI-NEC	All patients N = 297 (%)	High-volume n = 100 (%)	Medium-volume n = 105 (%)	Low-volume n = 92 (%)	*p* value
Etoposide + cisplatin	41 (13.8)	21 (21.0)	10 (9.5)	10 (10.9)	< 0.001
Etoposide + carboplatin	26 (8.8)	8 (8.0)	10 (9.5)	8 (8.7)	
Irinotecan + cisplatin	99 (33.3)	44 (44.0)	34 (32.3)	21 (22.8)	
Irinotecan + carboplatin	2 (0.7)	2 (2.0)	0 (0)	0 (0)	
Octreotide	20 (6.7)	2 (2.0)	8 (7.6)	10 (10.9)	
Everolimus	2 (0.7)	1 (1.0)	0 (0)	1 (1.1)	
S-1	19 (6.4)	2 (2.0)	7 (6.7)	10 (10.9)	
FP	9 (3.0)	2 (2.0)	6 (5.7)	1 (1.1)	
FOLFOX	15 (5.1)	1 (1.0)	6 (5.7)	8 (8.7)	
Other*	64 (21.5)	17 (17.0)	24 (22.9)	23 (25.0)	
Guideline-adherent proportion	**168 (56.6)**	**75 (75.0)**	**54 (51.4)**	**39 (42.4)**	

Regimens for P-NEC	All patients N = 127	High-volume n = 64	Medium-volume n = 35	Low-volume n = 28	
Etoposide + cisplatin	24 (18.9)	18 (28.1)	4 (11.4)	2 (7.1)	0.009
Etoposide + carboplatin	7 (5.5)	4 (6.3)	2 (5.7)	1 (3.6)	
Irinotecan + cisplatin	24 (18.9)	15 (23.4)	6 (17.1)	3 (10.7)	
Irinotecan + carboplatin	2 (1.6)	0 (0)	1 (2.9)	1 (3.6)	
Octreotide	15 (11.8)	7 (10.9)	3 (8.6)	5 (17.9)	
Everolimus	17 (13.4)	5 (7.8)	8 (22.9)	4 (14.3)	
Gemcitabine	9 (7.1)	4 (6.3)	3 (8.6)	2 (7.1)	
Other^†^	29 (22.8)	11 (17.2)	8 (22.9)	10 (35.7)	
Guideline-adherent proportion	**57 (44.9)**	**37 (57.8)**	**13 (37.1)**	**7 (25.0)**	

*FOLFOX* 5-fluorouracil, leucovorin and oxaliplatin, *FP* 5-fluorouracil plus cisplatin, *GEP* gastroenteropancreatic; *GI-NEC* gastrointestinal neuroendocrine carcinoma, *P-NEC* pancreatic neuroendocrine carcinoma, *S-1* tegafur-gimeracil-oteracil.

*Capecitabine + oxaliplatin + bevacizumab, S-1 + oxaliplatin + bevacizumab, capecitabine, capecitabine + cisplatin, capecitabine + cisplatin + trastuzumab, capecitabine + oxaliplatin, cisplatin, docetaxel + cisplatin + fluorouracil, docetaxel + cisplatin + S-1, doxorubicin + cisplatin + fluorouracil, cisplatin + S-1, cyclophosphamide + doxorubicin + vincristine, docetaxel, etoposide, fluorouracil, fluorouracil + irinotecan, fluorouracil + nedaplatin, fulvestrant, imatinib, irinotecan, miriplatin, S-1 + oxaliplatin, paclitaxel, panitumumab, pirarubicin, tegafur/uracil.

^†^Epirubicin, epirubicin + mitomycin, cisplatin, fluorouracil + irinotecan + oxaliplatin, fluorouracil + oxaliplatin, gemcitabine + S-1, sunitinib.

Significant values are in bold.

**Table 5:** Guideline-adherent proportion (%) calculated according to tumor classification, primary site, and hospital volume between 2013 and 2014.

	Guideline-adherent proportion	Hospital volume		
		High	Medium	Low
GI-NET	**58.1 (25/43)**	**77.8 (14/18)**	**50.0 (7/14)**	**36.4 (4/11)**
2013	58.8 (10/17)	85.7 (6/7)	50.0 (2/4)	33.3 (2/6)
2014	57.7 (15/26)	72.7 (8/11)	50.0 (5/10)	40.0 (2/5)
P-NET	**63.6 (42/66)**	**62.1 (18/29)**	**60.0 (12/20)**	**70.6 (12/17)**
2013	60.0 (15/25)	60.0 (6/10)	40.0 (4/10)	100.0 (5/5)
2014	65.9 (27/41)	63.2 (12/19)	80.0 (8/10)	58.3 (7/12)
GI-NEC	**56.6 (168/297)**	**75.0 (75/100)**	**51.4 (54/105)**	**42.4 (39/92)**
2013	48.5 (65/134)*	70.7 (29/41)	42.4 (25/59)	32.4 (11/34)
2014	66.3 (103/163)*	78.0 (46/59)	63.0 (29/46)	48.3 (28/58)
P-NEC	**44.9 (57/127)**	**57.8 (37/64)**	**37.1 (13/35)**	**25.0 (7/28)**
2013	36.4 (20/55)	44.8 (13/29)	40.0 (4/10)	18.8 (3/16)
2014	51.4 (37/72)	68.6 (24/35)	36.0 (9/25)	33.3 (4/12)

*GI-NEC* gastrointestinal neuroendocrine carcinoma, *GI-NET* gastrointestinal neuroendocrine tumor, *P-NEC* pancreatic neuroendocrine carcinoma, *P-NET* pancreatic neuroendocrine tumor.

*Guideline-adherent proportion differed significantly according to year.

Significant values are in bold.

Among 127 metastatic P-NEC patients, 24.4% of the patients received etoposide and cisplatin/carboplatin as a guideline-adherent first-line systemic therapy, and 20.5% received irinotecan and cisplatin/carboplatin as a first-line systemic therapy. Specifically, 25.2% of the patients received octreotide or everolimus. The guideline adherence proportion was 44.9%. Hospital volume was significantly associated with the guideline adherence proportion: 57.8% for high-volume hospitals, 37.1% for medium-volume hospitals, and 25.0% for low-volume hospitals (*p* = 0.009). Calculating by year, the guideline adherence proportions were 36.4% (20/55) in 2013 and 51.4% (37/72) in 2014 (*p* = 0.107, Table 5).

## Discussion

This study investigated guideline-adherent first-line systemic therapy regimens for metastatic GEP-NEN patients administered in the real-world setting using hospital-based cancer registry data and insurance claims-equivalent data in Japan. The proportion of GEP-NEN patients whose treatments adhered to the guidelines was 54.8%. To the best of our knowledge, this study is the first in the world to report on real-world selection of first-line systemic therapy regimens for metastatic GEP-NEN. We also evaluated the impact of publication of Japanese guidelines for GEP-NEN on the adherence to guidelines for first-line systemic therapy regimens.

For metastatic GEP-NET patients, the guideline adherence proportions were 58.1% for GI-NET and 56.1% for P-NET. A small but significant proportion of patients received fluoropyrimidine or gemcitabine systemic therapies for adenocarcinoma, which was the most common histology in the gastrointestinal tract and pancreas. Of note, the guideline adherence proportions tended to be correlated with hospital volume for metastatic GI-NET patients, but not for metastatic P-NET patients. Considering the moderate adherence to guideline treatment in general, a stricter compliance with guideline recommendations for metastatic GEP-NET is needed. However, physicians might have prioritized treatments for other malignancies, such as breast cancer, in these patients.

In contrast, GEP-NEC is generally a more aggressive tumor than GEP-NET^19–21^. Therefore, treatment for GEP-NEC is generally prioritized, even when other malignancies are present. And this speculation might affect improvement of the guideline adherence proportions for metastatic NEC patients in 2014. For metastatic GEP-NEC patients, the guideline adherence proportions were 56.6% for GI-NEC and 49.9% for P-NEC. The guideline adherence proportion was significantly correlated with hospital volume in both the GI-NEC and P-NEC patients. Overall, 6.7% of the metastatic GI-NEC patients received treatment for metastatic GI-NET (octreotide^13^ and everolimus^15,16^), and 14.5% of the metastatic GI-NEC patients received treatment for metastatic gastrointestinal cancer (S-1^22,23^, FP^24,25^ and FOLFOX^26–28^). Similarly, 25.2% received treatment for P-NET, and 7.1% received treatment for pancreatic cancer (e.g., gemcitabine). These non-adherent treatments might create very serious problems, especially in middle- or low-volume hospitals, as they could lead to a survival disadvantage arising from inappropriate treatment. Especially, prognosis of NEC remains poor^19,20^, accurate diagnosis and treatments are needed for metastatic NEC patients. Considering our results such as about 60% patients with GEP-NEN received systemic therapies in middle- or low-volume hospitals, standardization of treatments and centralization of patients might be needed for providing guideline-adhered treatment to advanced NEC patients.

At present, the appropriate standardization of first-line systemic therapy for metastatic GEP-NEN does not yet seem to have been achieved in Japan. To ensure that metastatic GEP-NEN patients can receive appropriate palliative systemic therapy, the development and dissemination of the guidelines on GEP-NEN or educational activities regarding treatments for GEP-NEN patients might be solutions. In contrast, when the guideline adherence proportion for GEP-NEC from before the announcement of the Japanese guidelines on the Internet in November 2013 was compared with that after the announcement in 2014, the proportion increased significantly, suggesting a possible impact of the guidelines on treatment selection.

The number of GEP-NEC patients who received first-line systemic therapy was numerically more than that of GEP-NEN patients in this study and past study showed same tendency, too^5^. The reason might be the difference in disease prognosis between GEP-NET and GEP-NEC; for example, GEP-NEC was an aggressive disease and was detected as an advanced stage. Therefore, the frequency of metastatic GEP-NEC patients received systemic therapy might be high.

This study had several limitations. First, the current study did not investigate the association between the guideline adherence proportion and treatment outcome because the linked data did not include survival data, such as the progression-free survival and overall survival periods. Secondly, because we could not exclude patients with multiple cancers, the guideline adherence proportions could have been underestimated or overestimated. An analysis using individual patient data might be useful for evaluating guideline-adherent drug therapies for tumors with a relatively good prognosis. Third, the hospital-based registries did not include locally assessed data for the Ki-67 index and differentiation, and there were only data for 2013 and 2014, which made in difficult to make evaluations based on the WHO classification or in other years. Moreover, the accuracy of local assessments of differentiation and the Ki-67 index were unclear. Therefore, it is possible that cases of NET-G3 were included in our NEC group. However, the standard treatments for NET-G3 were not established based on the results of clinical trials, and further investigations for NET-G3 are needed to decide guideline-recommended treatments. Fourth, the hospital-based registries did not include data on the date of pathological diagnosis. Therefore, some patients might have received palliative chemotherapy before diagnosis of GEP-NEN because of aggressive disease, leading to underestimation of the guideline adherence proportions. Finally, the second edition of the Japanese guidelines for GEP-NEN, published in 2019^29^, might enable a more appropriate dissemination of evidence-based medicine for metastatic GEP-NEN patients. Therefore, further research that includes detailed data on informative patient characteristics, such as disease status, anti-tumor activity of treatment, and survival outcome (for example, the NET registry [UMIN000016380], PROP-UP [UMIN000015976], and R-GENTE^21^) is needed.

In summary, this is the first report on real-world adherence with the Japanese guidelines for first-line systemic therapy regimens for metastatic GEP-NEN. In this study, about half of the patients with metastatic GEP-NEN received systemic therapy that was not recommended in the guidelines. Appropriate centralization of metastatic GEP-NEN patients and promotion of standardized treatments are warranted. Further studies, such as improving the guideline adherence proportions or evaluating the contribution of guideline-based treatments to survival benefit, are needed.

## Data Availability

Our data was permitted for use for only this study. The corresponding author (Chigusa Morizane) should be contacted if someone requests the data of this study.
