# Author Correction: The real-world selection of first-line systemic therapy regimen for metastatic gastroenteropancreatic neuroendocrine neoplasm in Japan

**DOI:** 10.1038/s41598-022-26509-z

**Published:** 2022-12-19

**Authors:** Shun Yamamoto, Naoki Sakakibara, Hidekazu Hirano, Chigusa Morizane, Yoshitaka Honma, Susumu Hijioka, Takuji Okusaka, Takahiro Higashi, Akira Kawai

**Affiliations:** 1 Department of Head and Neck, Esophageal Medical Oncology, National Cancer Center Hospital, Tokyo, Japan; 2 Department of Gastrointestinal Medical Oncology, National Cancer Center Hospital, Tokyo, Japan; 3 Division of Health Services Research, Institute for Cancer Control, National Cancer Center, Tokyo, Japan; 4 Department of Hepatobiliary and Pancreatic Oncology, National Cancer Center Hospital, 5-1-1 Tsukiji, Chuo-Ku, Tokyo, 1040045 Japan; 5 Department of Musculoskeletal Oncology and Rehabilitation, National Cancer Center Hospital, Tokyo, Japan

Correction to: *Scientific Reports* 10.1038/s41598-022-22718-8, published online 20 October 2022

The Funding section in the original version of this Article was omitted. The Funding section now reads:

“This work was supported in part by The National Cancer Center Research and Development Fund (28-A-16 and 31-A-14)”.

The original Article has been corrected.

